# Collective ordering of microscale matters in natural analogy

**DOI:** 10.1038/srep10790

**Published:** 2015-06-01

**Authors:** Sungsook Ahn, Sang Joon Lee

**Affiliations:** 1 Biofluid and Biomimic Research Center; 2 Department of Mechanical Engineering, Pohang University of Science and Technology, Pohang, 790-784, South Korea

## Abstract

Collective interaction occurs in many natural and artificial matters in broad scales. In a biological system, collective spatial organization of live individuals in a colony is important for their viability determination. Interactive motions between a single individual and an agglomerate are critical for whole procedure of the collective behaviors, but few has been clarified for these intermediate range behaviors. Here, collective interactions of microscale matters are investigated with human cells, plant seeds and artificial microspheres in terms of commonly occurring spatial arrangements. Human cancer cells are inherently attractive to form an agglomerate by cohesive motion, while plant chia seeds are repulsive by excreting mucilage. Microsphere model is employed to investigate the dynamic assembly equilibrated by an attraction and repulsion. There is a fundamental analogy in terms of an onset of regular pattern formation even without physical contact of individuals. The collective interactions are suggested to start before the individual components become physically agglomerated. This study contributes to fundamental understanding on the microscale particulate matters and natural pattern formation which are further useful for various applications both in academic and industrial areas.

Patterning is pervasive in nature from high order to complete randomness^1^,^2^. A perfectly ordered system shows characteristic spatial patterns, whereas a perfectly random system is unlikely to have determinant positional information. Many systems in nature actually occur between these two extremes. Collective behaviors occurring in almost every scales from animals to unicellular organisms^3^,^4^ as well as in inanimate particles^5^,^6^ are one important way of dynamic pattern formation. Biologically or artificially activated particulate systems exhibit characteristic swarm patterns that result from their dynamic interactions^7^,^8^. In terms of the pattern formation driven by collective behaviors, the local alignment across the population of motile individual results in an order over the length scales greater than the range of individual interaction. Collective behaviors of a colony naturally aim to advantageous purposes. In ecological systems, the degree to which individuals are aggregated or dispersed determines how a species uses resources, how it is used as a resource, and its reproductive biology^9^,^10^. Movement in animal groups is highly varied and ranges from seemingly disordered to coordinated motion aligned in flocks and schools. These social interactions are often thought to reduce risk from predators^11^. Considering that collective behaviors are optimized for a specific purpose, the nature-inspired spatiotemporal pattern formation usefully provide inspirations for a variety of product design methodology.

Certain analogies in various colonies can be made regardless of different system-specific character and scales. For example, collective animal behavior has been linked to understand zoomed-in interactions of tumor cell populations^12^,^13^. Social caterpillars and multicellular patterns of confluent human glioma cells have been analogically investigated even with their intrinsically different scales. There has been conceptual foundation in understanding collective cell interaction by exploiting analogies with the classical particulate systems based on density-dependent diffusivity. Collective behaviors are not necessarily dependent on the characteristics of the interacting individual components^14^. Statistical mechanics shows that large interacting systems undergo a change in population-level properties similar to physical systems such as liquid−gas phase transition. Generally applied universal pattern formations are suggested for diverse systems by the relation of analogy. On the other hand, biological phenomena is suggested to be explained based on the physical view point by analogy.

A sudden and spontaneous transition between an order and a disorder exists according to the density of interacting elements. Individual motions change from a relatively erratic to a correlated state over long distances or vice versa. For mass-migrating insects^15^ and keratinocytes^16^, such a transition has been experimentally validated. In a cellular system, collective cell migration in a contiguous cell sheet is characterized by physiological cooperation^17^,^18^, but the local transmission of physical forces in a group remain under dispute^19^,^20^. The transition from quasi-stable to stable aggregates exhibiting collective migration occurs by attractants and motility-inducing chemicals released from the cells. This transition results in the formation of various cell aggregates, such as tendrils, cone-like extensions^21^, strands, and columns^22^. The issues on the collective behaviors that occurs in nature are the mutual interaction among the individual components wherein a range of correlation is determined^23^,^24^. In continuous cell agglomerates, the continuum limit is determined as the onset point of cohesive correlation. On the other hand, between the isolated single cell and continuum limit of a physically contiguous cell sheet, the region where the cells are not in physical contact but in cooperation is not fully described yet.

In this study, collective behaviors of human cells, plant seeds and artificial microspheres are analogically investigated as representative natural systems. Human cancer cells are inherently attractive forming a continuous cell sheet. On the other hand, plant chia seeds are repulsive due to excreting mucilage until they are stabilized for sprouting. Surface-modified microspheres in a designed liquid medium undergo characteristic diffusive motions in a certain range along with the population. In the region between an isolated single individual and a continuum limit of a physically contiguous agglomerate, the correlation of inter-object distance distribution is identified. Spatial and temporal distance distributions are investigated in terms of pair correlation and dynamical heterogeneity to investigate the microscale particulate matters of both live and artificial systems.

## Results

### Human cancer cell distribution

**Figure 1:**
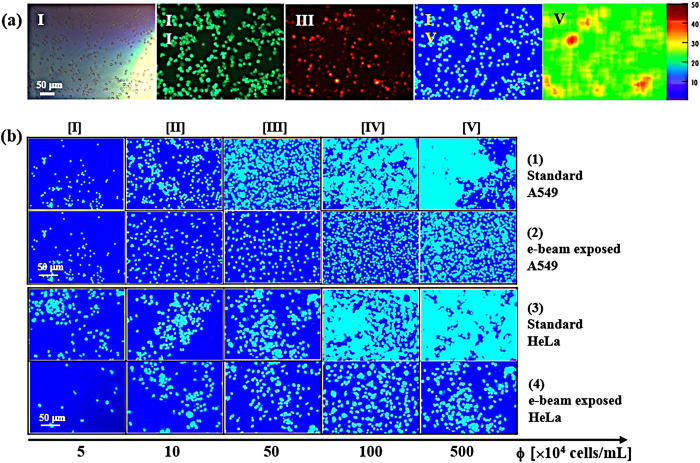
Cancer cell models. **(a)** Fluorescence images of representative *in vitro* A549 cancer cell model at a 5 × 10^5^  cells/mL. [I] typical optical microscopic image, [II] green fluorescence stained for actin shape, [III] red fluorescence stained for integrin motility, [IV] blue stain for nucleus, and [V] population density profile (blue to red indicates low to high density leveled by 50 degree). **(b)** Variation of cell distribution with increasing concentration from 5 to 500 (×10^4^ cells/mL) obtained from the nucleus stained images. Images for the standard A549, e-beam exposed A549, standard HeLa, and e-beam exposed HeLa cells are displayed from top to bottom.

Density-driven collective phenomena occurring in the cell colonies are investigated by typical human cancer cell lines. To obtain natural distribution of the cells on a substrate, surface activity, polarization of the cells and cell-substrate interaction are considered. To get reproducible tendency of the cell distributions, the density-controlled grown cells are dispersed on a SiN_3_ membrane and stabilized for the designed time at 37 °C and 5% CO_2_ condition. By this procedure, the natural distributions of the healthy cells are investigated. Figure 1(a) displays a representative image set of fluorescence-dyed A549 (adenocarcinomic human alveolar basal epithelial cells) cells at the population density (φ) of 10 × 10^4^ cells/mL stabilized for 18 hours (Supporting Information). Typical optical microscopy image of combined wavelength [I], actin labeling by phalloidin–fluorescein isothiocyanate stained in green [II], motility expression by Alexa Fluor 594 stained in red [III]. The nucleus positions are located by DAPI (4',6-diamidino-2-phenylindole) in sky blue against deep blue [IV]. The relative population density of the cells is color-coded by the scale bar in 50 levels [V]. Among the fluorescence images, nucleus-stained images are selected to quantify the cell-to-cell distance distribution at 18 hr stabilization as arranged in Fig. 1(b) for A549 and HeLa (cervical cancer cells). Typical snapshots of cell arrangements at the designed steady state reveal a change in cell-to-cell distance distributions along with φ from 5 [I] to 500 [V] (×10^4^ cells/mL). Cells of the same density (φ) are exposed to an electron beam (e-beam) (Supporting Information). The cell cluster growth is induced by the cell-to-cell attraction and collision, while cluster shrinkage is caused by the cells escaping from the edges and loosely connected parts. Typically, these growth and shrinkage compete and generate a definable cellular distribution. There is a specific concentration from which cluster formation becomes prominent for each cell type and properly introduced e-beam commonly retards the cluster formation for both cell types. Properly introduced e-beam induces physical modification of the cells by providing excess electrons to the cells thus inducing electrostatic interaction among the cells^25^,^26^,^27^. By e-beam exposure, the continuum limit of the cells moves toward a higher density for both cell types, indicating an increased repulsion among the cells^27^. It is suggested therefore that the cellular distance distributions are fairly dominated by the physical interactions.

**Figure 2:**
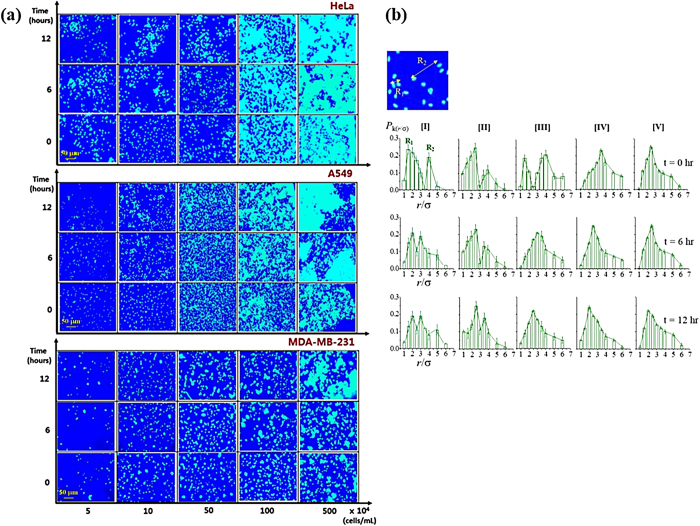
(**a**) From the nucleus stained images, variation of cell distribution with increasing density from 5 to 500 (× 10^4^ ells/mL) are obtained. Images for HeLa, A549, and MDA-MB-231 are displayed at 0, 12, and 18 hr of auto-arrangement of cells. **(b)** Representative distance distribution of the standard A549 cells. Population density is same as shown in Fig. 1C from [I] to [V] for 0, 12 and 18 hr equilibrium time.

To investigate the density-driven temporal behavior of the cell colonies, cervical cancer cells (HeLa), adenocarcinomic human alveolar basal epithelial cells (A549) and human breast cancer cells (MDA-MB-231) are prepared as typical human cancer cell lines. Cells are prepared in proper culture media, collected, concentrated, and then fluorescence-dyed (Supporting Information). After confirming the viability of fluorescence-dyed cells, nucleus-stained images are selected to quantify cell-to-cell distance distribution. The selected cell images are deployed from 5 to 500 (×10^4^ cells/mL) for 0, 12, and 18 hr of equilibrium time (Fig. 2(a)). All the cells slightly become more aggregated with time and the aggregation increases in the order: HeLa, A549, and MDA-MB-231. Cell distribution is diversified by the cell type and population density. There can be potential cell damage and image interference caused by the dye molecules and light exposure. Cell response is sensitive to many environmental factors thus there is a possibility that the cell cultures do not properly reflect the true behavior of cells but form noises. Therefore, for all the series of samples, the experiments are carefully controlled to obtain quantitative distance distribution determined only by the density-driven conditional factors while other factors are fixed.

The particulate interaction is quantified by the number ratio of the particulate objects (*P*_*k*_) placed in a particular distance *k* (number of particle pairs having a distance *k* divided by total counted particle pairs, *P*_*k*_ =*N*_*k*_/Σ*N*_*i*_). Thus *P*_*k*_ is determined as a probability of a cell to be in a distance *k* at time t. The described cluster dynamics is in contrast with cell cluster formation driven by differential cell adhesion and/or cell proliferation. The characteristic time for cell division is around 24 hours, thus the measured relaxation dynamics in this study occurs before the cell proliferation. Consequently, neglecting the cell proliferation, *P*_*k*_ is considered as a steady state value which depends on φ at a certain transition time. The clustering process evolves towards a dynamic equilibrium, where the process of cell cluster formation of a given size is balanced by the events in which clusters of this size disappear either by fusing with other clusters or by losing cells from the cluster body. Figure 2(b) exhibits a quantification of the distance distributions represented by the standard A549 according to the cell density at 0 hr, 12 hr and 18 hr stabilization. At a low population density in [I], the cell-to-cell distance exhibits a multimodal distribution reflecting inhomogeneity of the system. Along with an increase in the cell density, the initially disorganized cell-to-cell distance is organized into a unimodal distribution having one maximum point [IV]. Then this optimum distance (the peak position) decreases from [IV] to [V] by increasing the cell density. Because of the inherently attractive interaction among the cells, the cell-to-cell distance becomes closer along with the cell density and the stabilization time. In contrast to the onset of cohesion in collective cell behavior as an agglomerate^28^,^29^, noncontact collective behavior is suggested in this study: the individual cells have impact on each other so that their positions are determined by mutual interaction even though they are not in a physical contact. Under the optimum density condition, the cells are closely packed into continuous sheets. This continuous sheet has been the main issues of the studies focusing on the onset of collective and cohesive motion of the cells^28^.

### Plant chia seed distribution

**Figure 3:**
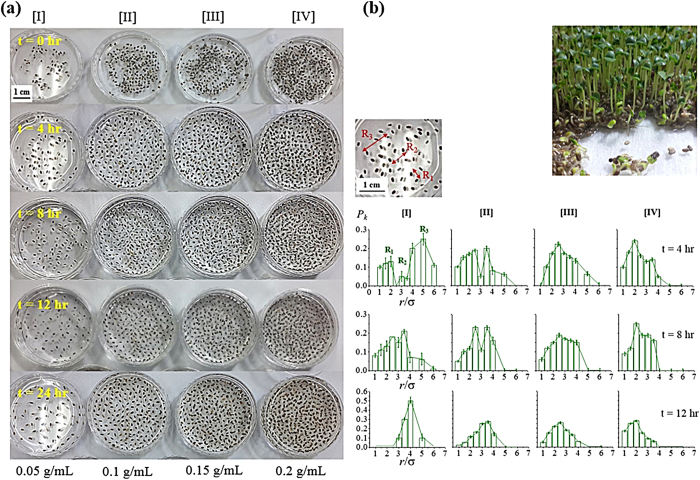
(**a**) Time- and concentration-dependent chia seed distribution. 0.05, 0.1, 0.15 and 0.2 g/mL of chia seeds are dispersed in water for and 0, 4, 8, 12 and 24 hr equilibrium. At the initial state at t = 0 hr, the seeds are not fully soaked in water, while the seeds are sprouted-out at 24 hr. **(b)** Quantitative distribution of the chia seeds according to the time and concentration shown in Fig. 2A.

Characteristic interactions between the particulate objects are found in the group of plant seeds. Chia seed (*Salvia hispanica* L.) is one example to excrete mucilage for self-protection against drying-out during their germination stages. Therefore, when the seeds are dispersed in water inherent repulsive interactions are expected to repel each other until the mucilage excretion stops. Simultaneously, by the mutual repulsive interaction among the seeds occurring in the overlapped spaces and by binding-out the seeds by viscous mucilage, attractive interaction is also contributed to the total force balance. Four concentrations of chia seeds dispersed in water and time-dependent seed-to-seed arrangements are displayed in Fig. 3(a). At the initial state at t = 0 hr, the seeds are not fully soaked in water while at the final state at t = 24 hr the seeds are sprouted-out. Quantitative graphical analysis of distance distribution of bare seeds (before sprouting) is displayed in Fig. 3(b) for stable positioning at the dispersion time of 4, 8 and 12 hr between the initial and the final states. Since the seeds are placed at the same condition (concentration, temperature, etc.), the amount of excreting mucilage is suggested to be similar generating isotropic repulsive interaction among the seeds. When the seed population is low and the amount of mucilage is small at the early stage, the distance distribution is diverse. However, with increased population from [I] to [IV] and more equilibrium time, the distribution becomes unimodal for all the population density. Mutual repulsion by the successively excreted mucilage followed by equilibrium generates isotropic and equally spaced seed-to-seed distance distribution. There is a similarity with the cancer cells in terms of the occurrence of unimodal after a certain diverse multimodal distance distribution. However, different from cancer cells, the position of the single peak is not deceasing by the time due to inherent repulsive interaction mediated by mucilage excretion. Both human cancer cell and plant chia seed systems aim at an optimized distance even though they are not in physical contact.

### Artificial microsphere model

**Figure 4:**
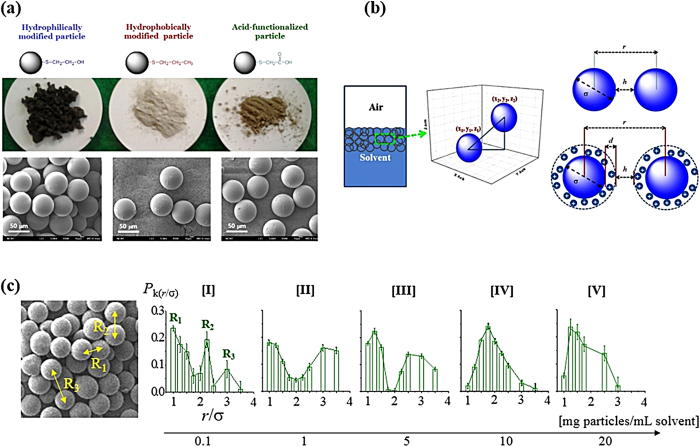
Microsphere models designed in this study . **(a)** Hydrophilic (−S−CH_2_−CH_2_−OH), hydrophobic (−S−CH_2_−CH_2_−CH_3_) and anionic (−S−CH_2_−COOH) particles are prepared. SEM image verifies the average diameter of 50 μm and narrow polydispersity of the particle size. **(b)** The designed particles are suspended in a solvent. The three-dimensional center-to-center distance, *r* = √[(*x*_1_ − *x*_2_) + (*y*_1_ − *y*_2_) + (*z*_1_ − *z*_2_)]^2^, of the selected particle pairs are estimated, while the surface-to-surface distance is denoted as *h*. The diameter of the particle is defined as σ. For charged anionic particle electrostatic layer *d* is determined. **(c)** Distance distribution of a representative −OH/H_2_O system according to the population density of the designed microspheres from 0.1 mg/mL [I] to 20 mg/mL [V].

Surface-functionalized hollow glass beads with average diameter of 50 μm are employed as a model system (Fig. 4(a)). A scanning electron microscopy (SEM) image confirms the narrow size distribution of the microspheres, reducing the polydispersity effect. Hollow glass beads of a fixed size (average diameter of 50 μm) with a narrow size distribution (~1% of size polydispersity) are surface-modified for chemical activity (Ψ) in a liquid media (Fig. 4(b)): −OH for hydrophilic, −CH_3_ for hydrophobic, and −COOH for anionic particles^30^. To minimize the molecular conformation effect three-carbon based molecules are employed, whereas a specific functional end-group is incorporated for chemical activity^30^. Water (H_2_O) and *n*-hexadecane (C_16_H_34_) are used as representative hydrophilic and hydrophobic solvents. The pH of the medium is controlled at 4, 7, and 10 to modify ionic strengths of the media. The *r* is the center-to-center distance of the particles, and σ is the particle diameter. For a charged particle, electrostatic layers of thickness *d* are formed by contribution of the Stern layer and the hydrodynamic layer (D_H_) around a particle. By X-ray CT, *r* value in each system is investigated to evaluate the particle-to-particle interaction.

The distance variation according to the compatibility of the microsphere in a dispersing medium is quantified by synchrotron X-ray computed tomography (X-ray CT) with a spatial resolution of 2 μm (Supporting Information). By matching the refractive index of the particles with the dispersing media, cooperative motions of particulate suspensions have been investigated by optical microscopy. On the other hand, synchrotron X-ray computed tomography (CT) has advantages in seeing through opaque objects in a noninvasive manner with effective phase contrast even without refractive index match. By the effective phase contrast generated by synchrotron X-ray of high coherence, hollow microspheres are visualized with high resolution^30^. Dispersion of particulate matters in fluids is controlled by adjusting the collective interactions induced by attractive van der Waals, repulsive electrostatic, and Brownian forces, as well as steric and hydrodynamic interactions. The van der Waals forces arise between all the molecules whether in air or in liquid, whereas naturally charged particles repel each other in aqueous solutions through electrostatic forces. Although sterically stabilized particulate matters are used as models to illustrate the equilibrium, transition, and nucleation, the microscopic time-dependent motions are affected by the suspending fluid^31^. As a kinetic phenomenon, interparticle interaction is quantitatively controlled by the compatibility of particles in fluids. Net force ***F***_net_(Ψ) applied to a microsphere in the designed system is evaluated by the relation; ***F***_net_(Ψ) = ***F***_buoyancy_ + ***F***_van der Waals attraction_ (Ψ) + ***F***_electrostatic repulsion_ (Ψ) + ***F***_other forces_ (Ψ). van der Waals and electrostatic forces are functions of the surface-functionality (Ψ), whereas buoyant force ***F***_buoyancy_ is not. ***F***_other forces_ (Ψ) is determined by the summation of the additional forces generated by the undulation in the local surface curvature of the interfaces. The microsphere systems employed in this study are dominated by electrostatic force than van der Waals force by designed surface modification and large size comparted to the typical colloidal systems (~1 nm to 1 μm). The microsphere colony is controlled to be overpopulated and pseudo-crystallized because of the buoyancy contribution based on φ. The buoyancy maximizes interparticle interactions according to the surface-modification and the collective behaviors of the designed systems are effectively induced in a long-range order. The interparticle distance of adjacent neutral microspheres with different functionalities varies based on the solvophilicity with a dispersing media.

As a representative system, the *P*_*k*_ of the −OH/H_2_O system is graphically quantified according to the population density of the particles from φ = 0.1 to 20 mg/mL (Fig. 4(c)). At a low φ condition [I], the distance distribution exhibits an apparent multimodal. The particles organized at a short distance (*r*/σ = 1) for physical contact and long distance (*r*/σ = 3 or larger) for cluster-to-cluster interaction. However, with an increase in the φ, the distance distribution goes to a unimodal having one main peak from [IV]. In terms of the occurrence of unimodal distance distribution after diverse multimodals, all the microscale systems investigated by human cancer cells, plant chia seeds and microsphere systems generate an optimized distance even without physical contact.

### Optimized distance distribution of μ-scale particulate matters

**Figure 5:**
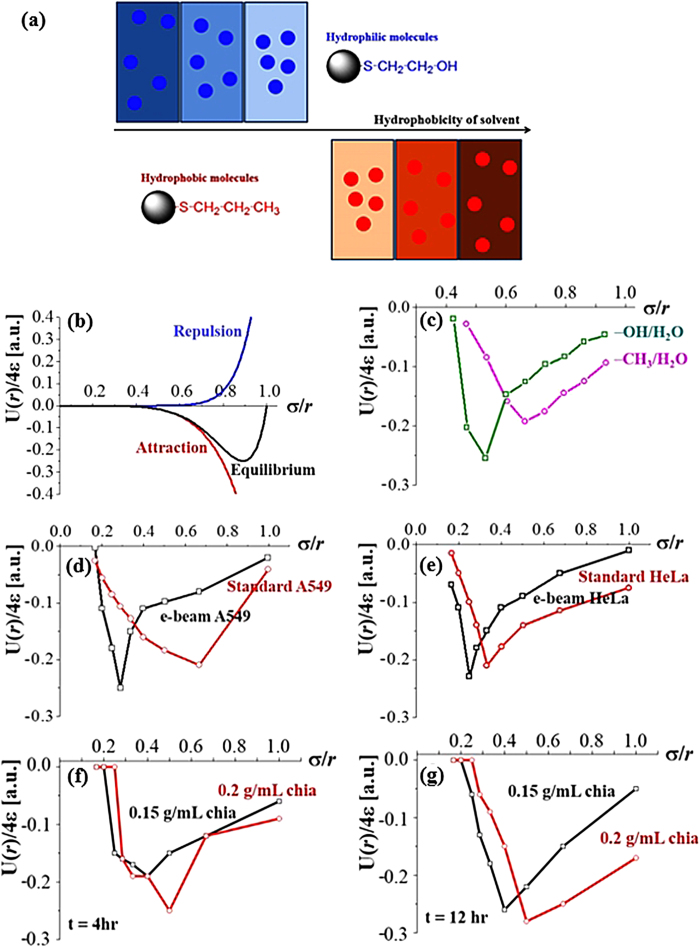
(**a**) Schematic illustration of the neutral particle interactions in solvents of varying solvability. The designed surface-modified microspheres are suspended in water and in an oleophilic liquid. When hydrophilic particles are suspended in hydrophilic media, the particles effectively disperse in the solvent because of the favorable interaction between the solvent and the particles, generating a longer inter-particle distance. In the same concept, hydrophobic particles are effectively dispersed in a hydrophobic medium. **(b)** Theoretical evaluation of the equilibrium between the repulsion and attraction energies of the particles by L-J potential. **(c)** Energy relation obtained from the experimental results of hydrophilic (−OH/H_2_O) and hydrophobic (−CH_3_/H_2_O) particles in water. **(d)** A549 cancer cells with and without e-beam exposure, and **(e)** HeLa cancer cells with and without e-beam exposure. **(f)** Chia seed distribution at 0.15 and 0.2 g/mL ate t = 4 hr equilibrium condition. **(g)** Chia seed distribution at 0.15 and 0.2 g/mL ate t = 12 hr equilibrium condition.

Regardless of the system-specific natural property in animal, plant and artificial particulate systems, there is an optimally organized distance distribution even without a physical contact according to the population density. Physically non-contacted but correlated individual particulate entities are explained by the typical force balance in long-range order. The energy associated with particle-to-particle is orders of magnitude larger than the energy associated with typical long range attraction/repulsion. Nonetheless, this energy balance is collectively expressed by the optimized distance distribution across the whole colony. The summated particle-to-particle distance equilibrated in a whole system generates a certain optimum distance in a colony. By balancing the repulsive and attractive forces, the interparticle interactions in a liquid medium reaches an equilibrium. In a typical particulate behavior in a media, the interparticle distance of the particles of a high compatibility with the dispersing solvent becomes long, while the particles of low compatibility are not effectively dispersed in the solvent media (or aggregated) (Fig. 5(a)). Therefore, for each particle−solvent combination, the particle-to-particle distance is stabilized within a certain range, reflecting inherent compatibility of the system. Lennard-Jones (L-J) potential is typically employed to describe the equilibrated interaction between the neutral particle pairs^32^: U(*r*) = 4ε[(σ/*r*)^12^ − (σ/*r*)^6^], where ε denotes the depth of the energy well, *r*^−12^ the electrostatic repulsion, and *r*^−6^ the long-range attraction induced by the van der Waals force (Fig. 5(b)). The free energy reaches a primary minimum after a monotonic decrease, and then reaches a bound state if there is no prominent energy barrier. Particle association is reversible depending on the depth of the energy potential and on the processes that occur in the aggregated state. The depth of the U(*r*) represents the stability of the interparticle interaction, while the radial position of the minimum energy, (σ/*r*)_m_ indicates the optimum interparticle distance. The energy level is experimentally evaluated by the relation,


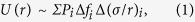


considering a portion of the particles placed in a distance *i* away from the minimum energy state at the physical contact (*r* = σ). The interaction energy of the particles, U(*r*) is quantified by the *P*_*k*_. The positional energy difference per particle pair between the zero-energy state at contact (U(*r*) = 0) and a certain equilibrium state at the position of (σ/*r*)_*i*_ is used to evaluate the differences in the force Δ*f*_*i*_ and in the inverse normalized distance Δ(σ/*r*)_*i*_. Thus, the energy is proportional to the number ratio of corresponding particle pairs placed at a distance *k* (*P*_*k*_). The theoretical equilibrium curve of attraction and repulsion is reproduced by the L-J potential (Fig. 5(b)). An energy well is formed by the existence of the minimum U(*r*)/4ε at a certain (σ/*r*)_m_ position, this corresponds to the maximum particle number ratio, (*P*_*i*_)_m_ obtained experimentally at 10 mg particle/mL solvent condition (Fig. 5(c)). A deeper energy at a smaller (σ/*r*)_m_ value is observed in hydrophilic particles suspended in water (−OH/H_2_O) compared with the hydrophobic particles (−CH_3_/H_2_O). This indicates that effective dispersion of hydrophilic particles in water generates almost unimodal distribution in the particle-to-particle distance. The experimentally evaluated potential energy of A549 (Fig. 5(d)) and HeLa (Fig. 5(e)) are displayed with and without e-beam exposure at the cell population of 5 ×10^4^ cells/mL. For both cell types, the e-beam exposed cells exhibit smaller (σ/*r*)_m_ value with slightly deeper energy for both cell types. The difference between the standard and e-beam exposed cells is more prominent for A549 than HeLa. This indicates a characteristic physical property of the cells to be more susceptible to the e-beam introduction. On the same concept, time- and population-dependent chia seed distributions are evaluated in Fig. 5(f,g) at t = 4 hr and t = 12 hr equilibrium condition. The definable distance distribution of the plant system also shows well-fitted patterns to the relation of L-J potential.

### General description of the optimized distance distribution

The microscale particulate systems in human cellular systems and plant seed systems show a similarity with the designed artificial microsphere systems, where the force balance is well fitted to the L-J potential-like patterns. For typical particulate systems, attraction induced by the van der Waals force and the electrostatic repulsion are equilibrated. Based on the observed phenomena, the biological systems also can be explained based on the force balance between the attraction (*K*_attraction_) and the repulsion (*K*_repulsion_) even though cause of force for each system are different. For the cellular system, the attractive forces are generated by the cell signaling to induce cellular cohesion while repulsion is generated by electrostatic interaction. For a plant chia seeds, the attraction is induced by the stickiness of the mucilage while the repulsion is induced by excretion of the mucilage. Therefore, generalized formalism is suggested here by introducing system-specific constant (*E*), as well as repulsive and attractive force balance which are proportional to the inverse power of the normalized distance (*R*),


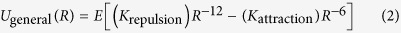


### Radial distribution function, g(*r*) of the cells

Macroscopic properties of bulk fluids are obtained in terms of radial distribution function, g(*r*) which describes the probability of observing a particle that is located at a distance (*r*) away from a given particle center by the relation of 4*πr*^2^ρ*g*(*r*), where ρ is the number density. The g(*r*) is a local measure of the resemblance of a particulate matter distribution to a uniform one. As the first particle is placed into an empty container, this particle can be located at any position. Therefore, all the locations are likely equal, corresponding to a uniform probability distribution. However, the next particle can be located anywhere except within a particle diameter (σ) distance from the first particle. This process continues until all *N* particles have been added to the container. At each successive step of this process, the free area available to the next added particle is less than that of the previous particle. Therefore, the particle location is not random but is dependent on the positions of all the preceding particles. As the area fraction of the particles in the system increases, this excluded area effect becomes more pronounced^31^. The influence of one particle on its neighbors also extends to increasingly greater distances, causing the g(*r*) to deviate from unity well beyond the nearest neighbors of a central particle. Although the excluded volume effect is a fundamental type of interaction, many other types of particle-to-particle interactions exist. Among those effects, electrostatic interactions may significantly influence the observed g(*r*).

**Figure 6:**
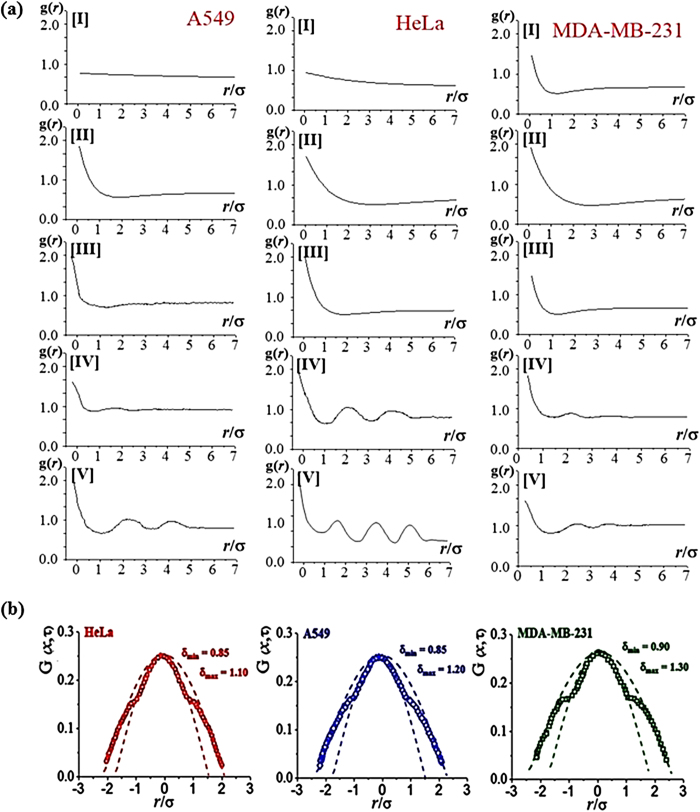
(**a**) Pair correlation function g(r) of designed cancer cells according to the population density from 5 [I] to 500 [V] (× 10^4^ cells/mL) at 12 hr equilibrium time. **(b)** Modified Gaussian distribution functions *G*(*x*,τ) of designed cancer cell systems are quantitatively expressed by the degrees of deviation using δ_max_ and δ_min_.

The g(*r*) of the cell colonies of A549, HeLa, and MDA-MB-231 (human breast cancer cells) are prepared (Fig. 6(a)). After confirming the viability of fluorescence-dyed cells, nucleus-stained images are selected to determine the g(*r*) deployed from 5 [I] to 500 [V] (×10^4^ cells/mL) condition for 18 hr of aging time. From the obtained g(*r*), the influence of one cell affecting the behavior of another within a specific distance range is determined by the end point of the fluctuation as a correlation length. This correlation length is the distance required for the g(*r*) to level off to unity, over which a cell influences others. Hence any correlation disappears above such distance. The g(*r*) achieves unity when the cell distribution is uniform. This finding indicates that no correlation exists among the cells, thus the behavior of one cell does not influence any other. Meanwhile, the g(*r*) values of greater/less than unity imply regions of positive/negative correlation in which the influence of the cells at the origin is deviated. The g(*r*) is approximately one for all the positions in the cell image at low population density of the cells. However, the g(*r*) considerably differs from one at high population density of the cells with fluctuations above the proper *r*/σ value. The influence of one cell on its neighbors extends to increasingly greater distances, causing the g(*r*) to deviate from unity well beyond the nearest neighbors of a reference point. This explanation is consistent with the experimentally observed fluctuation that increased from unity as the population density of the cells increases from [I] to [V]. With the increased population density, a HeLa cell influences its neighbors up to six times away from the unit distance (*r*/σ = 1). This correlation length is five times for A549, and four times for MDA-MB-231. The short correlation length is found for less aggregated cells observed in the fluorescence images in Fig. 2(a).

### Modified Gaussian distribution, G(x ,τ) of the cells

Time-dependent cellular arrangement affected by each cell behaviors are investigated by observing the cell behaviors for 18 hrs. Molecular transport is generally dominated by Fickian diffusion, in which the mean square displacement is proportional to the elapsed time. Brownian motion assumes that fast-moving small molecules follow random walk^33^, which leads to the statistical mechanism as a Gaussian-distributed stochastic temporal series^34^. For sufficiently long-time separation, random walk dynamics leads to Gaussian distribution and the diffusion follows Fickian by central limit theorem^35^. Nonetheless, recent observations in many systems exhibit deviation from the Gaussian without a long-time separation, even though the displacement distribution follows Fickian^36^,^37^,^38^. The designed cells are equilibrated at a given population density of 5 × 10^5^ cells/mL according to the temporal variation. The modified Gaussian distribution is evaluated by the degree of deviation (δ) by the relation: G(*x*,*τ*) ∝ exp(−*x*^2^τ/2δ^2^), where *x* is the displacement, and τ is the elapsed time (Fig. 6(b)). Collective phenomena, hydrodynamic behavior, self-organization, local equilibrium and propagation of chaos are the main features in the evolution of many-body systems. In this study we consider stochastic interacting particle systems where some of these effects are expected. The main feature of the particulate model we consider is that in such continuum limit the generator splits into two parts, one is larger than the other. As a consequence, two time scales are distinguished; in the first the evolution seems to be ruled by the main larger part of the generator, while the effect of the smaller one enters into play in the second longer time scale. The modified Gaussian fittings inserted in each case are marked by the dotted lines. The results reflect that the observation of G(*x*,τ) is reasonably described by the sum of two Gaussians: A wide fitting (δ_max_) represents the fast-moving fraction of the cells, whereas a narrow fitting (δ_min_) expresses the slow-moving portions. Therefore, the population of the cells consists of prominently faster and slower subpopulations than those expected from a single Gaussian. The difference between these two subpopulations, |δ_max_ − δ_min_| increases in the order: HeLa, A549, and MDA-MB-231. By that sequence, the cells are less aggregated with longer cell-to-cell distance, suggesting a highly dynamical heterogeneity that is similarly observed in colloidal hard-sphere suspensions^39^.

### Radial distribution function, g(*r*) of the microspheres

**Figure 7:**
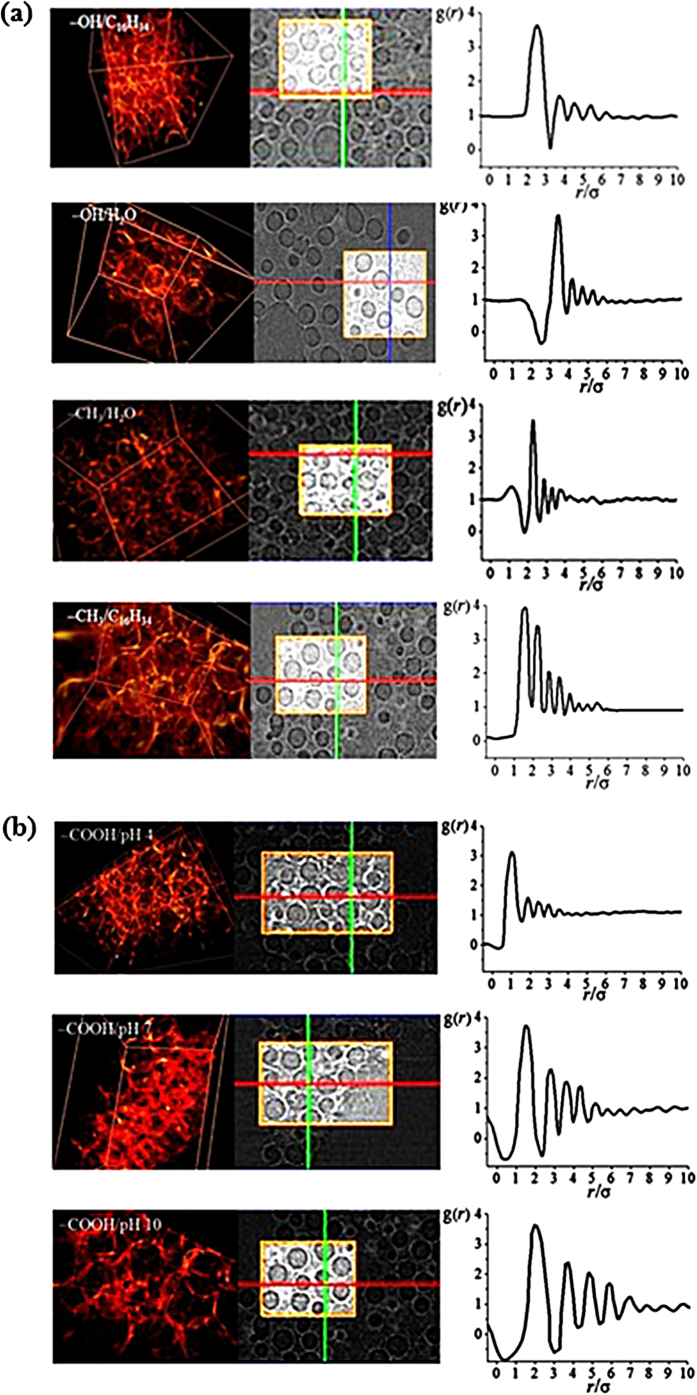
In the left 3D image shots (left) and 2D sectional images (right) obtained by synchrotron X-ray CT are arranged . On the other hand, in the right graphs, corresponding pair correlation function g(*r*) is displayed for **(a)** hydrophilic particles suspended in water (−OH/H_2_O) and in hexadecane (−OH/C_16_H_34_) and hydrophobic particles in hexadecane (−CH_3_/C_16_H_34_) and in water (−CH_3_/H_2_O) and for **(b)** anionic particles suspended in pH 4 (−COOH/pH 4), pH 7 (−COOH/pH 7) and pH 10 (−COOH/pH 10).

Representative 3D image shots (Fig. 7(a) left images) and 2D sectional images (Fig. 7(a) middle images) obtained by synchrotron X-ray CT (a spatial resolution of 2 μm, Supporting Information) are arranged for neutral and charged particles. For each image set, corresponding g(*r*) of neutral particle systems are arranged at the right as a function of the normalized interparticle distance (*r*/σ) at a fixed population density of ρ = 5 mg particles/mL solvent. The center-to-center distance (*r*) of −OH/H_2_O system is relatively longer than its counterpart that is dispersed in less compatible solvent, −OH/C_16_H_34_. Given the longer *r* of the particles dispersed in compatible solvents (for −OH/H_2_O and −CH_3_/C_16_H_34_ systems), the first main peak of *g*(*r*) results in a higher *r*/σ value than their counterparts in less compatible solvents (for −OH/C_16_H_34_ and −CH_3_/H_2_O systems). Nonetheless, the correlation length continues to approximately six to seven times for all the systems without prominent differences in each solvent–particle combination. The results of the anionic (−COOH) microspheres dispersed in pH 4, 7, and 10 solutions are shown in Fig. 7(b). With increasing pH, the interparticle distance increases by the electrostatic repulsion of more effectively charged functional group (−COO^−^) at high pH condition than neutralized form (−COOH) at low pH condition. The correlation length prominently becomes longer at high pH condition as induced by effective electrostatic repulsion. Therefore, non-ionic particles in neutral solvents do not generate prominent difference in the correlation length of each particle–solvent combination. The range of the particle interaction is similar regardless of particle–solvent type. Furthermore, the charge effect on the particles generates effectively extended pair correlation distance, which is sensitively differentiated by the degree of charge according to the pH condition of the media.

### Modified Gaussian function, G(x,τ) of the microspheres

**Figure 8:**
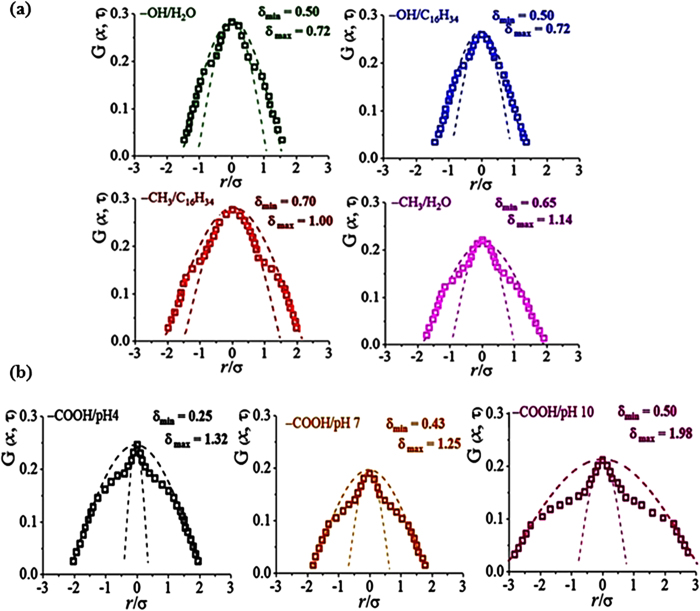
Modified Gaussian distribution functions, G(x,τ), quantitatively expressed by the degrees of deviation, δ_max_ and δ_min_ for (**a**) hydrophilic particles suspended in water (−OH/H_2_O) and in hexadecane (−OH/C_16_H_34_) and hydrophobic particles in hexadecane (−CH_3_/C_16_H_34_) and in water (−CH_3_/H_2_O) and for (**b**) anionic particles suspended in pH 4 (−COOH/pH 4), pH 7 (−COOH/pH 7) and pH 10 (−COOH/pH 10).

The designed microspheres are equilibrated in adequately long timescales at a fixed population density (Fig. 8(a)). A wide (δ_max_) and narrow (δ_min_) fittings show deviation from a single Gaussian, depending on the particle–solvent combination. Rather than hydrophilic particles (−OH), hydrophobic particles (−CH_3_) in neutral solvents exhibit prominent difference in those two δ_max_ and δ_min_, indicating highly dynamical heterogeneity. Prominently, charged particle systems exhibit high deviation that expresses fast- and slow-moving subpopulations, thereby reflecting a high dynamical heterogeneity. Therefore, charged particles exhibit significant dynamical heterogeneity (Fig. 8(b)) and broader distance distribution with longer correlation length as previously observed. In comparison with the aforementioned cellular behaviors, MDA-MB-231 of broad distance distribution thus less aggregated system displays high dynamical heterogeneity among the designed cells in this study. At this point, charged microsphere systems show closely similar distribution to those of cell colonies exhibiting differentiated correlation lengths. This result suggests that the time-dependent distance distribution of the cells is mainly induced by the electrostatic effects.

### Cooperative association models employed for cellular aggregation

From the aforementioned results in this study, it is suggested that the cell-to-cell distance distributions are dominated by the physical interactions including attraction-repulsion equilibrium similarly occurring in the artificial microsphere systems. Therefore, the cluster formation of the colloidal systems is relevantly employed to describe the mechanism of the cellular organization. In the bottom-up cluster formation, individual cells are aggregated from a dilute medium. From the point of dynamic particulate interactions, flocculation is considered against the concept of irreversible association or fusion (coagulation or coalescence). Three typical cooperative association processes^40^ are considered to describe the aggregation processes of the interactive cells: isodesmic model, phase separation model, closed-association model. The systems are considered to be reversible before reaching an equilibrium. In a stepwise addition without additional interactions, a monomer S is added to an aggregate S_*n*–1_. This induces S_*n*_ aggregation by introducing equilibrium constant, K_*n*_.


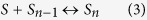



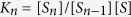


In the isodesmic model, each individual is continuously added to the aggregate and K_*n*_ is independent of the number *n*. There is no abrupt onset in a narrow population density range, and an aggregate size is not definable. By contrast, the phase separation model assumes that the aggregates with a large *n* are dominant. The standard free energy of an aggregate of a certain size is determined by the energy relation,


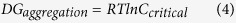


Considering that the critical aggregation concentration (*C*_critical_) indicates the minimum size of the stable cluster, the phase separation model describes the aggregation of a certain size. Finally, in the closed-association model, *n* individuals form aggregates simultaneously. Thus, only aggregates composed of *n* individuals and monomers exist,


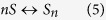



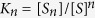


**Figure 9:**
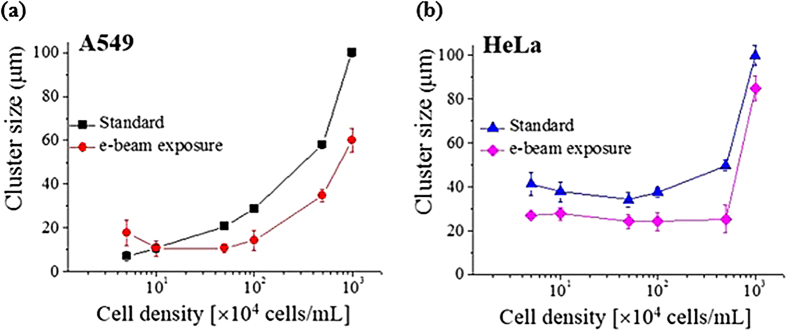
Variation of the cluster size of the cells according to the population density (φ) of the cells ranging from 5 to 1000 × 10^4^ cells/mL for (**a**) A549 and (**b**) HeLa cells with and without e-beam exposure. For A549 the isodesmic model is suitable reflecting a continuous association of individual cells. By contrast, for HeLa the phase separation model is adoptable.

The average cluster size (S_*n*_) is experimentally obtained from the discontinuous aggregates of the individual cells. The results are semi-logarithmically plotted according to the cell density of each system (Fig. 9). The cluster size of both standard and e-beam exposed A549 continuously increases with increasing population density until a continuum is formed. Therefore, for the A549 association procedure, the isodesmic model is suitable reflecting a continuous association of individual cells. HeLa exhibits rather discontinuous increase in the cluster size. It forms a large chunk from a low cell density condition and then the cluster size retains almost similar even with the increase in the cell density. And then it shows rather abrupt increase in their size from a certain point. Therefore, from the perspective of collective behavior, the phase separation model is adoptable for the association procedure of the HeLa, for which a certain size of aggregates composed of *n* individuals is suggested to be dominant.

The observed results indicate that the cooperative association models can be employed to describe the cluster formation of the cells. Even though the reason why isodesmic model is suitable for A549 colony while phase separation model is better to describe the HeLa aggregate is not clear at this moment, the inherent cell property can be proposed to explain the observed results. In many experiments, A549 typically floats on top of the media, while HeLa typically sticks on the wall and the bottom of the container. Therefore, rather individualistic A549 cells assemble one-by-one, while sticky HeLa form a group all at once. Properly applied e-beam induces the characteristic interaction between the cells due to modified electrostatic interactions^25^,^26^,^27^. In our results, A549 is observed to be more sensitive to the physical property change induced by e-beam introduction thus to be more repulsive each other electrostatically. These results do not explain directly how the cells are aggregated in different association kinetics, but provide some reasonable clues why the cell aggregate patterns are differentiated depending on the cell type. The physical modification of the cells by e-beam exposure does not change this cell-specific intrinsic association mechanism but only induces a shift in the absolute cell density value to reach a continuum limit.

## Discussion

The current results demonstrate quantitative measurement of microscale interactions of live and inanimate particulate matters, which are eminently plausible in nature. The human cells, plant seeds and artificial microsphere interactions in the intermediate ranges between a single individual and an agglomerate exhibit a significant analogy in terms of an optimized distance distribution after a multimodal diversity. The collective property in a colony is described by the equilibrium between long-range attractive and repulsive interactions. The live systems involve non-equilibrated and self-propelled particulate matter, while artificial microsphere system is in an equilibrium influencing their dynamic assembly. The short-range attractive interaction that arises from cell-to-cell adhesion mediated by cadherin and short range cell-to-cell elastic repulsion of two cells pushing against each other are the main cellular interactions reaching an equilibrium. The cell-to-cell interaction is not simply limited to the typically known short-range interactions but includes long-range interaction expressed by optimized distance distribution in a whole colony. The long-range patterns are general for cellular, plant seeds and microsphere systems which are suggested to be induced by the summation of the stronger short-range interactions occurring in a collective way rather than weaker long-range interactions. This generates a characteristic pair correlation, g(*r*) that covers up the several times of the cell diameter.

Even though biological systems are composed of integrated forces, live and inanimate systems exhibit significant analogy in terms of spatial and temporal distribution. Spatial and temporal distance distributions are investigated in terms of pair correlation, and dynamical heterogeneity by employing radial distribution function, g(*r*) and modified Gaussian distribution function, G(*x*,τ) to analyze the microscale particulate matters of both live and artificial systems. The live systems are inherently non-equilibrated and self-propelled systems. This defining characteristic is not supposed to be overlooked in their dynamic assembly and cluster formation. On the other hand, the artificial microsphere is an equilibrated system with no activity or self-propulsion. Nonetheless, the two live and artificial systems exhibit prominent analogy. The function of dynamics in the intermediate stage in a colony is not fully explored yet. But it is suggested here that the biological systems are prominently dominated by the physical interactions similar to the artificial particulate systems. Therefore, microscale biological systems are suggested to be explained by the physical interaction similarly occurring in the artificial particulate systems.

In conclusion, this study demonstrates the collective interaction of microscale particulate systems in natural analogy. The representative natural systems of human cells, plant seeds and artificial spheres in microscale, already start collective interactions before the individual components are physically agglomerated. These are generally expressed by an optimized distance distribution determined by equilibrated force balance. The present results demonstrate the characteristic collective behaviors occurring in microscale and quantitative analogy between biological and artificial systems, which are plausible in nature. The observed results shows that individual cell-to-cell interaction also shows characteristic and systematic distribution tendency against the concept of typically reported cell agglomeration behavior. These microscale biomimetic system might contribute to open a new way for the modelling-based study and to elucidate various yet-explained naturally occurring phenomena both for live and artificial systems.

## Methods

### Cell model preparation

HeLa cells (human cervical carcinoma cells, ATCC) and MDA-MB-231 cells (human breast cancer cells, ATCC) are cultured in DMEM (Dulbecco’s modified eagle’s media, Invitrogen). A549 cells (human lung carcinoma cells, ATCC) are cultured in RPMI-1640 media (Invitrogen). Both cell culture media contain 10% fetal bovine serum and 1% penicillin streptomycin. HUVECs (human umbilical vein endothelial cells, Invitrogen) are cultured in Medium 200 (Invitrogen) with the addition of Low Serum Growth Supplement (LSGS) containing 20% fetal bovine serum and 1% penicillin streptomycin. Cells are cultured in a humidified atmosphere with 5% CO_2_ at 37 °C. Trypsin (0.25%)/EDTA solution is used to detach the cells from the culture flask. The detached cells are pelleted with slow centrifugation and naturally resuspended in their culture media at six different concentration conditions from 5 to 500 (×10^4^ cells/mL). The density-controlled cell solutions are loaded on a flat SiN_3_ membrane to get reproducible distribution tendency. Then the cells are stabilized in a monolayer for 12 and 18 hr to get stability followed by washing-off by de-ionized water. For the selected cells, e-beam is applied for 2 sec for which cells are not damaged but change their physical property prominently.

### Fluorescence dyeing of the cells

After the cells are naturally distributed on a SiN_3_ membrane, the cells are instantly fixed for fluorescence dyeing with 4% paraformaldehyde in DPBS solution (Dulbecco’s Phosphate-Buffered Saline, Invitrogen) for 20 min at room temperature and then unstable cells are washed-off three times with DPBS. Antibody anti-hCD51/61 (5 μg/mL) for αvβ3 integrin conjugated with PE (R&D systems, Minneapolis, MN, USA) are overlaid on the cell surface overnight at 4 °C and washed using PBS solution 3 times for 1 min each. The cells are in red color by fluorescence microscopy. Secondary antibody Alexa Fluor 594 donkey anti-mouse IgG antibody (10 μg/mL) is loaded overnight at 4 °C and washed using PBS solution 3 times for 1 min each to stain integrin of the cells. Phalloidin-fluorescein isothiocyanate (FITC) (5 μg/mL) was added to stain actin for 30 minutes at room temperature and then washed using PBS 3 times for 1 min each. This provides green colors to the cells by fluorescence microscopy. Actin participates in many important cellular processes, including muscle contraction, cell motility, cell division and cytokinesis, vesicle and organelle movement, cell signaling, and the establishment and maintenance of cell junctions and cell shape. Many of these processes are mediated by extensive and intimate interactions of actin with cellular membranes. Vertebrates have three main groups of actin isoforms: α-actin, β-actin, and γ-actin. The α-actins found in muscle tissues are a major constituent of contractile apparatuses. The β-actin and γ-actin coexist in most cell types as components of cytoskeleton, and as mediators of internal cell motility. DAPI (4',6-diamidino-2-phenylindole) (100 μg/mL) is added to stain nucleus for 5 min at room temperature followed by washing with PBS solution 3 times for 1 min each. This generates blue colors in the cells by fluorescence microscopy. By staining nuclei of the fixed cells, the locations of the individual cells are identified.

### Fluorescence microscopy and image analysis

The cell images are obtained with a fluorescence microscopy (Zeiss Axiovert 200 fluorescence microscope) equipped with 20× phase-contrast objective lens (NA = 0.4) and an AxioCam MRc CCD camera. An X-Cite 120 Q excitation light source (120 W mercury vapor short arc lamp), filter sets for Alexa Fluor 594, Fluorescein isothiocyanate (FITC), and DAPI solutions are used for fluorescence imaging. The microscope is automatically operated and the images are acquired by Axiovision 4.8.2 software (Carl Zeiss). The measurements are performed by computer algorithm. And among four types of images (Figure S2) nucleus stained images is selected for distance quantification. Even with the decreased distance the resolution is enough to decide the center-to-center distance of the nucleus due to high enough intensity of the fluorescence and clear boundary. In addition, under the given condition one cell level image is possible thus normalized distance (*r*/σ, σ is the diameter of a single cell) can be reasonably obtained.

### E-beam application

The experiments were performed at the Pohang Neutron Facility (Pahang, South Korea). The materials include an electron linac, a water-cooled Ta target, and an 11 m long time-of-flight path. The electron linac consists of a thermionic RF-gun, an alpha magnet, four quadrupole magnets, two SLAC-type accelerating sections, a quadrupole triplet, and a beam-analyzing magnet. A 2 m long drift space was added between the first and second accelerating sections to insert an energy-compensation magnet or a beam-transport magnet. The overall length of the linac was about 15 m. The RF gun has one cell cavity with a dispenser cathode of 6 mm in diameter. The RF gun produces electron beams of 1 MeV, 300 mA, and 1.5 ms. Four quadrupole magnets were used to focus the electron beam in the beam transport line from the thermionic RF gun to the first accelerating section. The installed quadrupole triplet between the first and second accelerating sections was used to focus the electron beam during the transport to the experimental beam line at the end of the linac. The maximum available RF power from a SLAC 5045 klystron is 45 MW because of the peak power limitation of the pulse modulator. The fed RF power to the RF gun was 3 MW. The beam energy was 50 MeV. The measured beam currents at the entrance of the first accelerating section and at the end of the linac were 100 and 40 mA, respectively. The width of an electron beam was 3 μs pulse, and the pulse repetition rate was 10 Hz. The electron beam was about 20 mm in diameter at the beam profile monitor in front of the target. Given the known resistance at the Ta target (2 Ω), the total electrons that reached the samples can be calculated in relative scales by the voltage versus time data. The measured energy spread was less than 1%. The e-beam was directly applied to the designed cell solutions in Whirl-Pak^®^ sample bag (B00679WA model, Nasco, USA) for duration times of 0.5, 1, 1.5, 2, 3, 4, 5, 10, and 15 sec conditions are tried. For all the samples, the amount of electrons was confirmed to be similar without significant deviations. And among that condition, 2 sec is selected because it shows a prominent e-beam effect without any physical damage of the cells. The viability of the cells are confirmed by the fluorescence images. At that condition, there is no abnormal deformation of the cell shape but only the distance distributions are changed. This prominent distance change is considered by the e-beam effect while the cell viability is maintained.

### Surface-modified hollow microspheres model

Monodispersed silver-coated hollow glass microspheres with a diameter of 53 μm are purchased (Cospheric LLC, Santa Barbara, USA). Thionic compounds are strongly bound to the Ag element. To reduce the molecular conformation effect, only three-carbon based molecules are designed, whereas a specific functional end-group is incorporated for chemical activity (Ψ): 1 M aqueous ligand solution of 5 mL is added to modify the surface properties of the microspheres (0.1 g microsphere/1 mL DI water) by mercaptoethanol (−S−CH_2_−CH_2_−OH, hydrophilic particle) and 1-propanethiol (−S−CH_2_−CH_2_−CH_3_, hydrophobic particle).

### Synchrotron X-ray tomography

Experiments are conducted at the 6D beamline of the third generation synchrotron radiation source of the Pohang Accelerator Laboratory (Pohang, Korea). The radiation source comprises bending magnets with an average energy of 21.5 keV at 2.5 GeV. To minimize thermal damage to the sample due to over exposure to X-ray radiation, a 1000   μm-thick silicon attenuator that cuts off photon energy below 10 keV is installed. The size of the beam illuminating the sample is fitted to the field of view (FOV) using a slit module to avoid unnecessary sample exposure to the X-rays. A mechanical shutter was used to expose the sample to the X-ray beam when X-ray images were captured.

### Chia seed preparation

Chia seeds (*Salvia hispanica* L.) are dispersed in tap water at the designed four concentrations (0.05, 0.1, 0.15 and 0.2 g/mL) to observe the seed-to-seed distance distribution. Experiments are conducted at the 6D beamline of the third generation synchrotron radiation source of the Pohang Accelerator Laboratory (Pohang, Korea). The radiation source comprises bending magnets with an average energy of 21.5 keV at 2.5 GeV. To minimize thermal damage to the sample due to over exposure to X-ray radiation, a 1000 μm-thick silicon attenuator that cuts off photon energy below 10 keV is installed. The size of the beam illuminating the sample is fitted to the field of view (FOV) using a slit module to avoid unnecessary sample exposure to the X-rays. A mechanical shutter was used to expose the sample to the X-ray beam when X-ray images were captured.

## Additional Information

**How to cite this article**: Ahn, S. & Joon Lee, S. Collective ordering of microscale matters in natural analogy. *Sci. Rep.* **5**, 10790; doi: 10.1038/srep10790 (2015).

## Supplementary Material

Supplementary Information
