# Supplementary material for: Collective ordering of microscale matters in natural analogy
Source: Sci Rep. 2015 Jun 1;5:10790. doi: 10.1038/srep10790 (PMC4450545; doi:10.1038/srep10790)

**Collective ordering of microscale matters in natural analogy**

Sungsook Ahn^1,2^, Sang Joon Lee^1,2^ *

^1^Biofluid and Biomimic Research Center, ^2^Department of Mechanical Engineering,

Pohang University of Science and Technology, Pohang, 790-784, South Korea.

*CORRESPONDING AUTHOR.

Tel: +82-54-279-2169; Fax: +82-54-279- 3199; E-mail: sjlee@postech.ac.kr

**Supporting Information**

**Cell model preparation.** HeLa cells (human cervical carcinoma cells, ATCC) and MDA-MB-231 cells (human breast cancer cells, ATCC) are cultured in DMEM (Dulbecco’s modified eagle’s media, Invitrogen). A549 cells (human lung carcinoma cells, ATCC) are cultured in RPMI-1640 media (Invitrogen). Both cell culture media contain 10% fetal bovine serum and 1% penicillin streptomycin. HUVECs (human umbilical vein endothelial cells, Invitrogen) are cultured in Medium 200 (Invitrogen) with the addition of Low Serum Growth Supplement (LSGS) containing 20% fetal bovine serum and 1% penicillin streptomycin. Cells are cultured in a humidified atmosphere with 5% CO_2_ at 37 °C. Trypsin (0.25%)/EDTA solution is used to detach the cells from the culture flask. The detached cells are pelleted with slow centrifugation.

The cells are fluorescence dyes (see below) and naturally resuspended in their culture media at different population density (φ) from 5 to 500 (×10^4^ cells/mL). The density-controlled cells are monolayered on a flat SiN_3_ membrane to get reproducible distribution tendency. Then the cells are stabilized in a monolayer for 4 hours to get stability followed by washing-off by de-ionized water. For the selected cells, e-beam is applied for 5 sec. After the cells are naturally distributed on a SiN_3_ membrane, the samples are instantly fixed with 4% paraformaldehyde in DPBS solution (Dulbecco's Phosphate-Buffered Saline, Invitrogen) for 20 min at room temperature and then unstable cells are washed-off with DPBS.

*Fluorescence dyeing.* Antibody anti-hCD51/61 (5 μg/mL) for αvβ3 integrin conjugated with PE (R&D systems, Minneapolis, MN, USA) are overlaid on the cell surface overnight at 4 °C and washed using PBS solution 3 times for 1 min each. The cells are in red color by fluorescence microscopy. Secondary antibody Alexa Fluor 594 donkey anti-mouse IgG antibody (10 μg/mL) is loaded overnight at 4 °C and washed using PBS solution 3 times for 1 min each to stain integrin of the cells.

Phalloidin-fluorescein isothiocyanate (FITC) (5 μg/mL) was added to stain actin for 30 minutes at room temperature and then washed using PBS 3 times for 1 min each. This provides green colors to the cells by fluorescence microscopy. Actin participates in many important cellular processes, including muscle contraction, cell motility, cell division and cytokinesis, vesicle and organelle movement, cell signaling, and the establishment and maintenance of cell junctions and cell shape. Many of these processes are mediated by extensive and intimate interactions of actin with cellular membranes. Vertebrates have three main groups of actin isoforms: α-actin, β-actin, and γ-actin. The α-actins found in muscle tissues are a major constituent of contractile apparatuses. The β-actin and γ-actin coexist in most cell types as components of cytoskeleton, and as mediators of internal cell motility.

DAPI (4',6-diamidino-2-phenylindole) (100 μg/mL) is added to stain nucleus for 5 min at room temperature followed by washing with PBS solution 3 times for 1 min each. This generates blue colors in the cells by fluorescence microscopy. By staining nuclei of the fixed cells, the locations of the individual cells are identified.

*Fluorescence microscopy and image analysis.* The cell images are obtained with a fluorescence microscopy (Zeiss Axiovert 200 fluorescence microscope) equipped with 20× phase-contrast objective lens (NA = 0.4) and an AxioCam MRc CCD camera. An X-Cite 120 Q excitation light source (120 W mercury vapor short arc lamp), filter sets for Alexa Fluor 594, Fluorescein isothiocyanate (FITC), and DAPI solutions are used for fluorescence imaging. The microscope is automatically operated and the images are acquired by Axiovision 4.8.2 software (Carl Zeiss).

To get the spatial distribution of the cells from the raw images captured by an optical microscopy, digital image processing procedures are taken. At first, it is determined how to calculate the cell distribution. Some pictures are hard to count the cell-like pattern [III] below, because the boundaries of the cell are hard to be distinguished. Since direct cell counting is not determinant in getting the cell distribution, the number of pixels is counted to evaluate this the number distribution of the cells. The selected 20 sample images are statistically analyzed to get the number show average pixels of the cells. The size of each image used in this experiment is 800 (√800 × √800) pixels. Next, the region of the cells is determined. Images are painted sky-blue if the cell is located, dark-blue if not located in this experiment. Using MATLAB, the selected images are converted to 2-D array data. By analyzing this array, the region of the cells is simply identified. Then, the pixels are counted in a square field. By taking a region of 255 × 255 (2^8^) pixels. At each point, we count how many cells are located in the specific region. Counting the number of cells is simplified by using 2-D convolution method with a high-pass filter of 255 × 255 matrix filled with 1. Finally, depending on the number of the cells, the images are colored. It is able to get an image which is small number of cells into green, and large number into red. [II] and [IV] images show the spatial distribution of the cells generated by this method. If there is only 0~5 cells in the region of 255 × 255 pixels, the pixels are filled with green color, and if the cells are more than 45 cells, the pixels are filled with red color. Each color is divided into 50 levels.

**[I] [II] [III] [IV]**


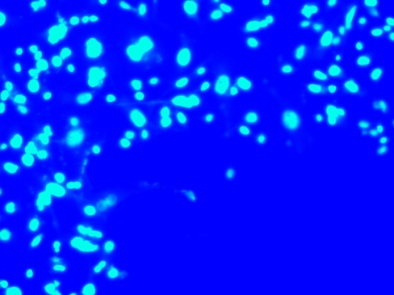

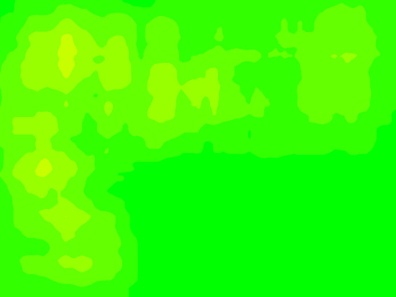

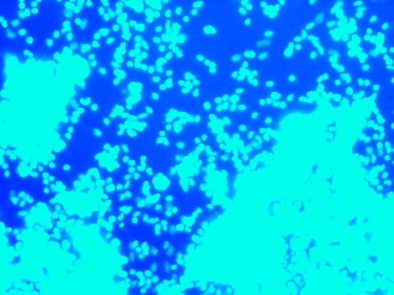

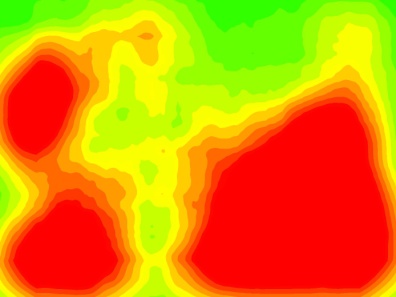

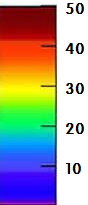


**Figure S1.** Image analysis procedure for monpolayered cell colonies.

**
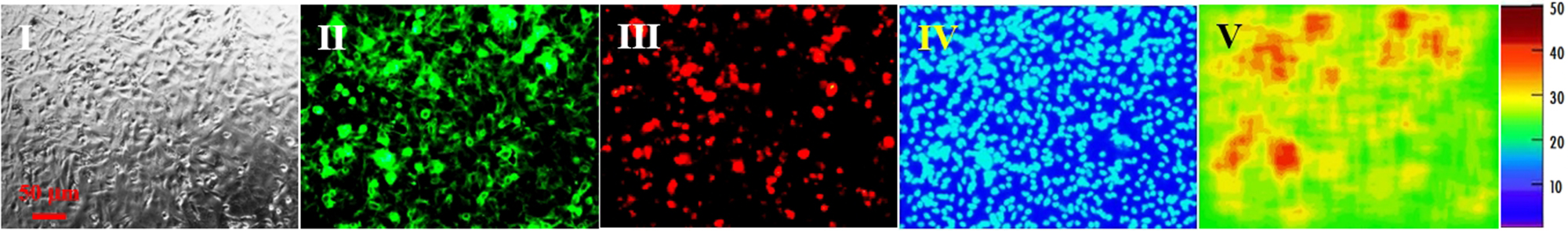
**

**Figure S2.** The cells are resuspended in the culture media at the controlled population density (φ), monolayered on SiN_3_ membrane to get reproducible distance distribution pattern (Supporting Information). Fig. 1A displays a representative image set of fluorescence-dyed A549 cells at the population density of 1×10^7^ cells/mL: typical optical microscopy image of combined wavelength [I], actin labeling by phalloidin–fluorescein isothiocyanate stained in green [II], motility expression by Alexa Fluor 594 stained in red [III]. The nucleus positions are located by DAPI (4',6-diamidino-2-phenylindole) colored in sky blue [IV]. The relative population density of the cells is color-coded [V]. After confirming the viability of the cells by fluorescence, nucleus-stained images [IV] are employed to quantify the cell-to-cell distance distribution.

**Synchrotron X-ray tomography.** Experiments were conducted at the 6D beamline of the third generation synchrotron radiation source of the Pohang Accelerator Laboratory (Pohang, Korea). This radiation source comprises bending magnets with an average energy of 21.5 keV at 2.5 GeV. To minimize thermal damage to the sample due to over-exposure to X-ray radiation, a 1000 μm-thick silicon attenuator that cuts off photon energy below 10 keV was installed to attenuate the white beam. The size of the beam illuminating the sample was fitted to the field of view (FOV) using a slit module to avoid unnecessary sample exposure to the X-rays. A mechanical shutter was used to expose the sample to the X-ray beam when X-ray images were captured. A scintillator made of a CdWO_4_ crystal was placed 10 cm behind the test sample to transform the X-ray into visible light. The phase contrast two-dimensional (2D) X-ray images were recorded using a charge-coupled device (CCD) camera that capture 4008 × 2672 pixels (VM-11M5, Vieworks). The FOV generated by a 10× objective lens attached in front of the camera, was approximately 3.5 mm × 2.3 mm.

**Figure S3.** Schematic diagram of the experimental set-up for the synchtron X-ray computed tomography (X-ray CT).

**
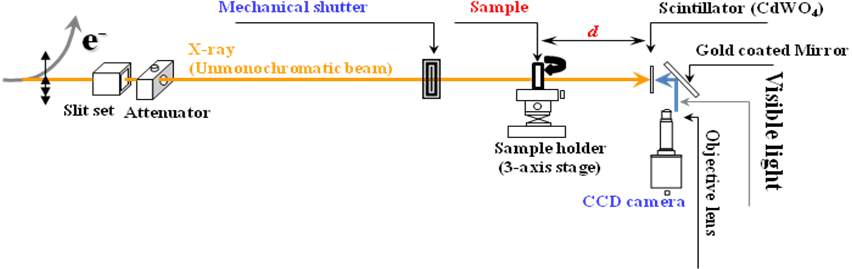
**

**Surface-modified hollow microspheres model.** Monodispersed silver-coated hollow glass microspheres with a diameter of 53 μm are purchased (Cospheric LLC, Santa Barbara, USA). The apparent color and the stickiness of the dried particles are varied depending on the surface-functionalization. Representative scanning electron microscopy (SEM) images show narrow size distribution which demonstrates small polydispersity effect. For the preparation of the samples for X-ray CT, hollow borosilicate glass capillaries (ID 1.00 mm, OD 1.20 mm, length 100 mm; CM Scientific Ltd, UK) are employed, in which the designed microspheres in a selected solvent are loaded at a concentration of 0.1 g microsphere/1 mL solvent (excluding the ligand mass). Five samples of the same particle−solvent combination are prepared to investigate the time-dependent interparticle distance. To obtain the distance distribution, 300 particle pairs at least 100 μm away from the container tube wall are selected to remove the wall effect for each sample. Five samples of the same composition are measured for a statistical average

**
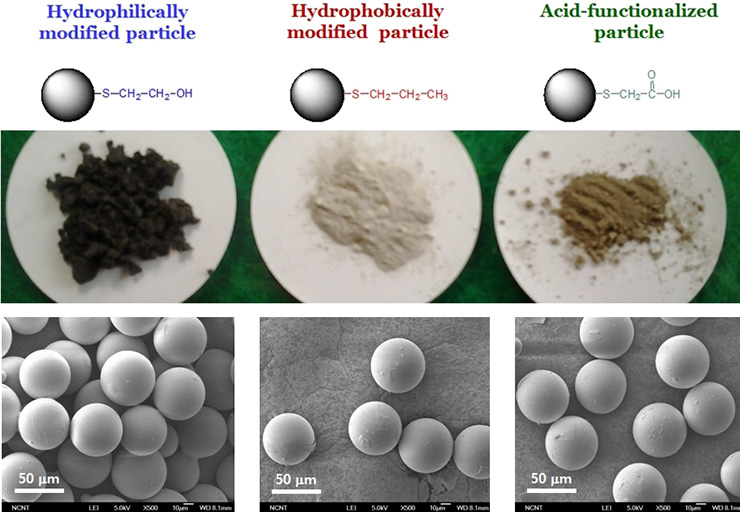
**

**Figure S4.** Chemical structures and the typical picture of the three different surface-modified microspheres: hydrophilic, hydrophobic and acid-functionalization. The average diameter of the designed hollow microspheres is 53 μm. Representative scanning electron microscopy (SEM) images of the particles are shown in the bottom line.

**Figure S5. Interparticle interaction analysis.** From the three-dimensional X-ray CT images (Fig. 2B), the net force ***F***_net_ (Ψ) applied to a microsphere in the designed system is evaluated; ***F***_net_ (Ψ) = ***F***_buoyancy_ + ***F***_van der Waals attraction_ (Ψ) + ***F***_electrostatic repulsion_ (Ψ) + ***F***_other forces_ (Ψ). van der Waals and electrostatic forces are functions of the surface-functionality (Ψ), whereas buoyant force ***F***_buoyancy_ is not. ***F***_other forces_ (Ψ) is determined by the summation of the additional forces generated by the undulation in the local surface curvature of the interfaces. The microspheres employed in this study are dominated by electrostatic force than van der Waals force by the designed surface modification and larger size than typical colloidal systems. The microsphere colony is controlled to be overpopulated and pseudo-crystallized because of the buoyancy contribution based on the population density (ρ, number of particles per volume). The buoyancy maximizes interparticle interactions according to the surface-modification and the collective behaviors of the designed systems are effectively induced in a long-range order. **(A)** Schematic illustration of the neutral particle interactions in the solvents of varying solvability. When hydrophilic particles are suspended in hydrophilic media the particles are effectively dispersed in a solvent due to favorable interaction between the solvent and particles. On the same concept, hydrophobic particles are effectively dispersed in a hydrophobic medium. **(B)** Schematic illustration of the acid-functionalized particle interaction in the solvents of varying pH. When acid-functionalized particles are suspended in acidic condition particles are less effectively dispersed in a solvent due to neutralized form of the acid-functionalized particle (COOH). On the same concept, acid-functionalized particles are effectively dispersed in a high pH medium because of effective charge formation on a particle (COO^−^).

**A**

**
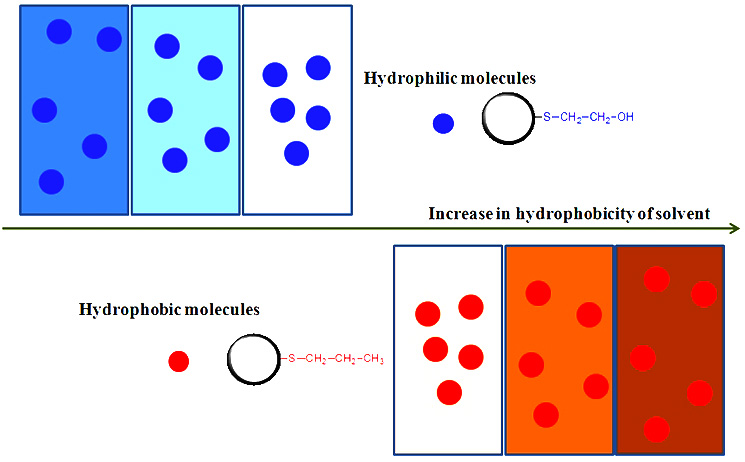
**

**B**

**
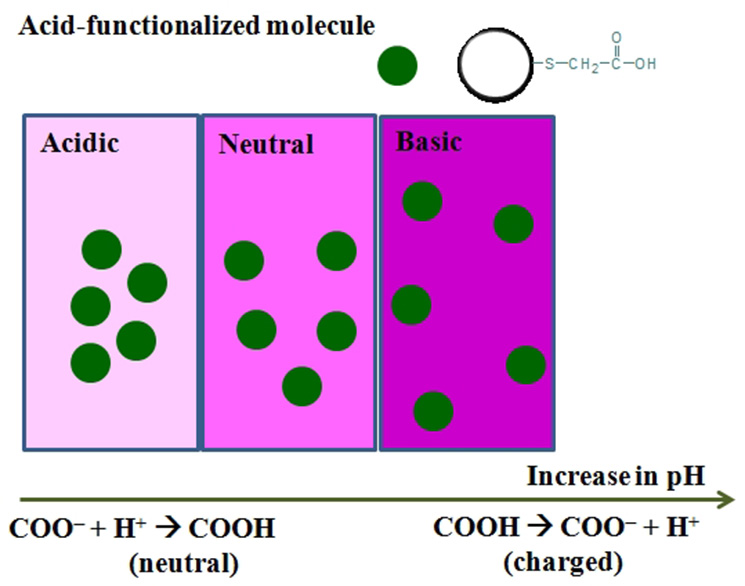
**

**Figure S6. X-ray CT image anaylysis.** Schematic illustration of the digital image analysis procedures for X-ray CT reconstruction. The procedures follow from A to D in consecutive order.


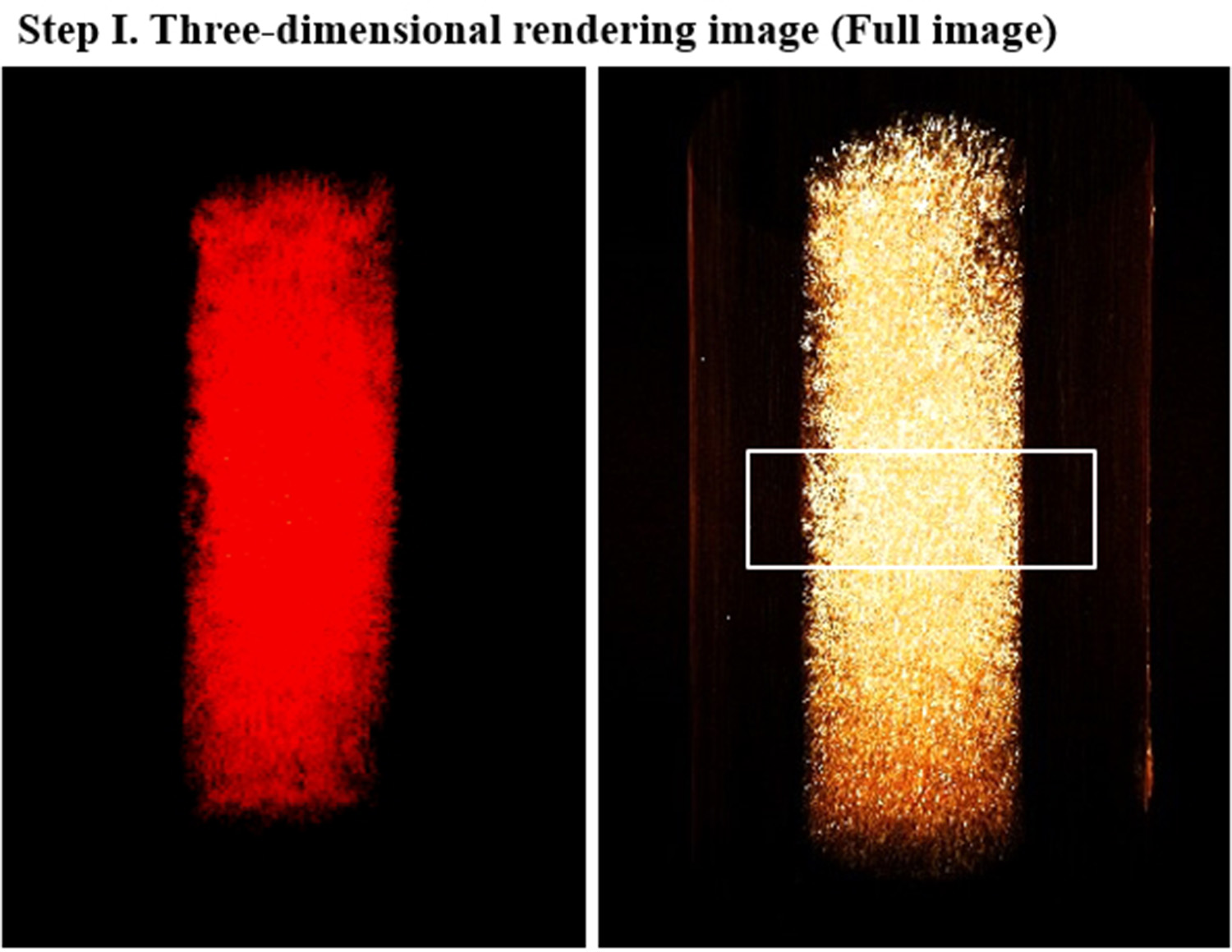


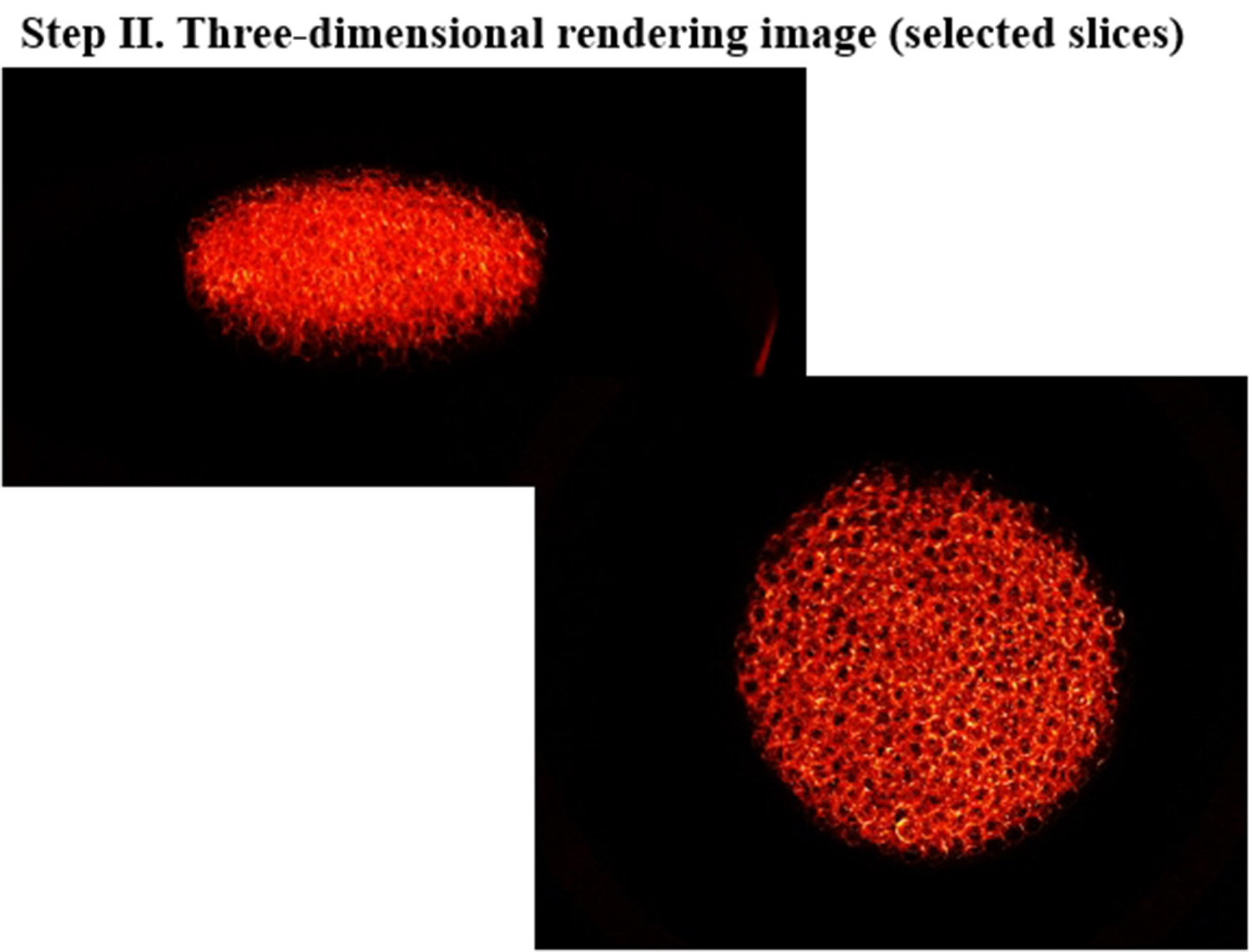


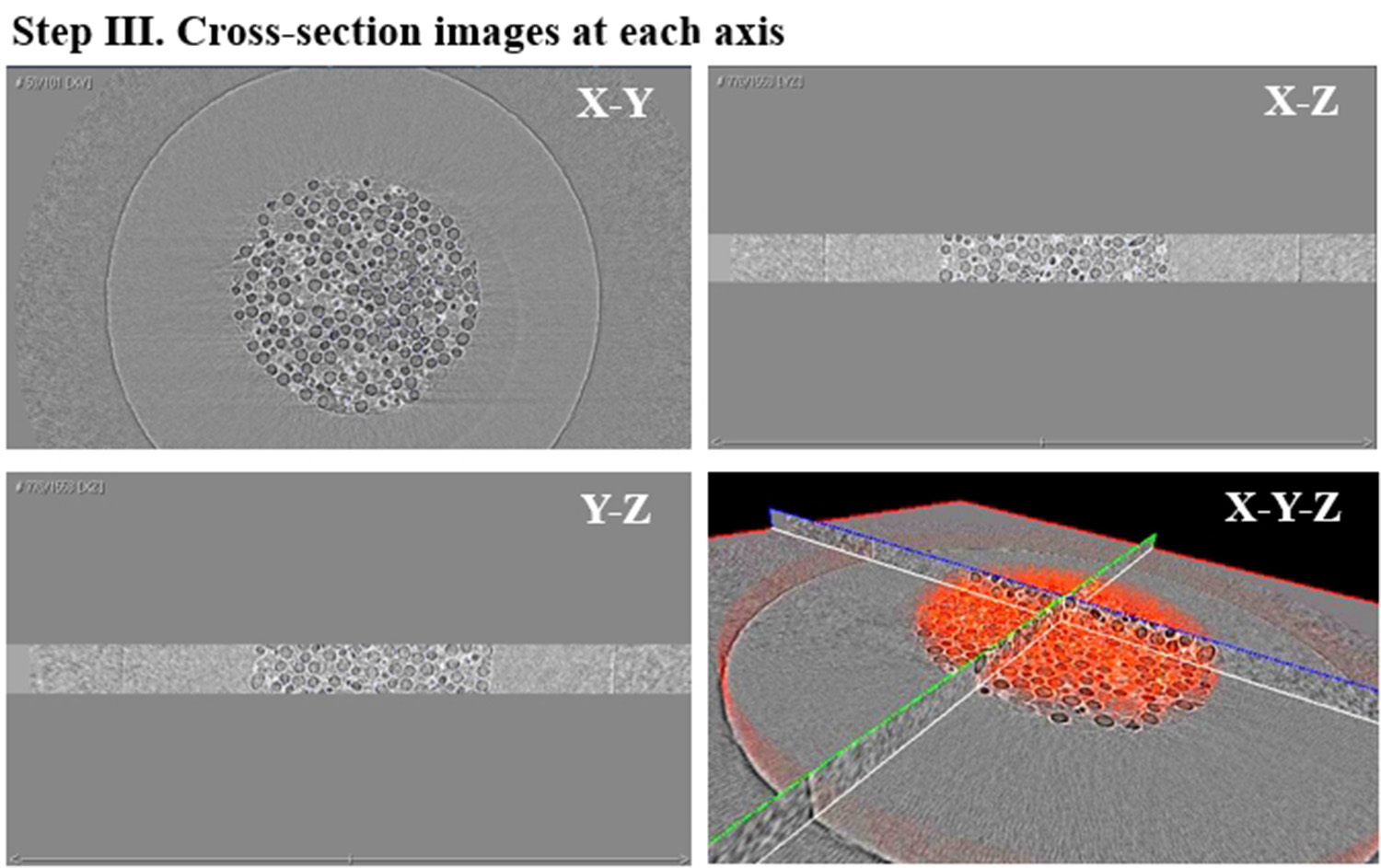


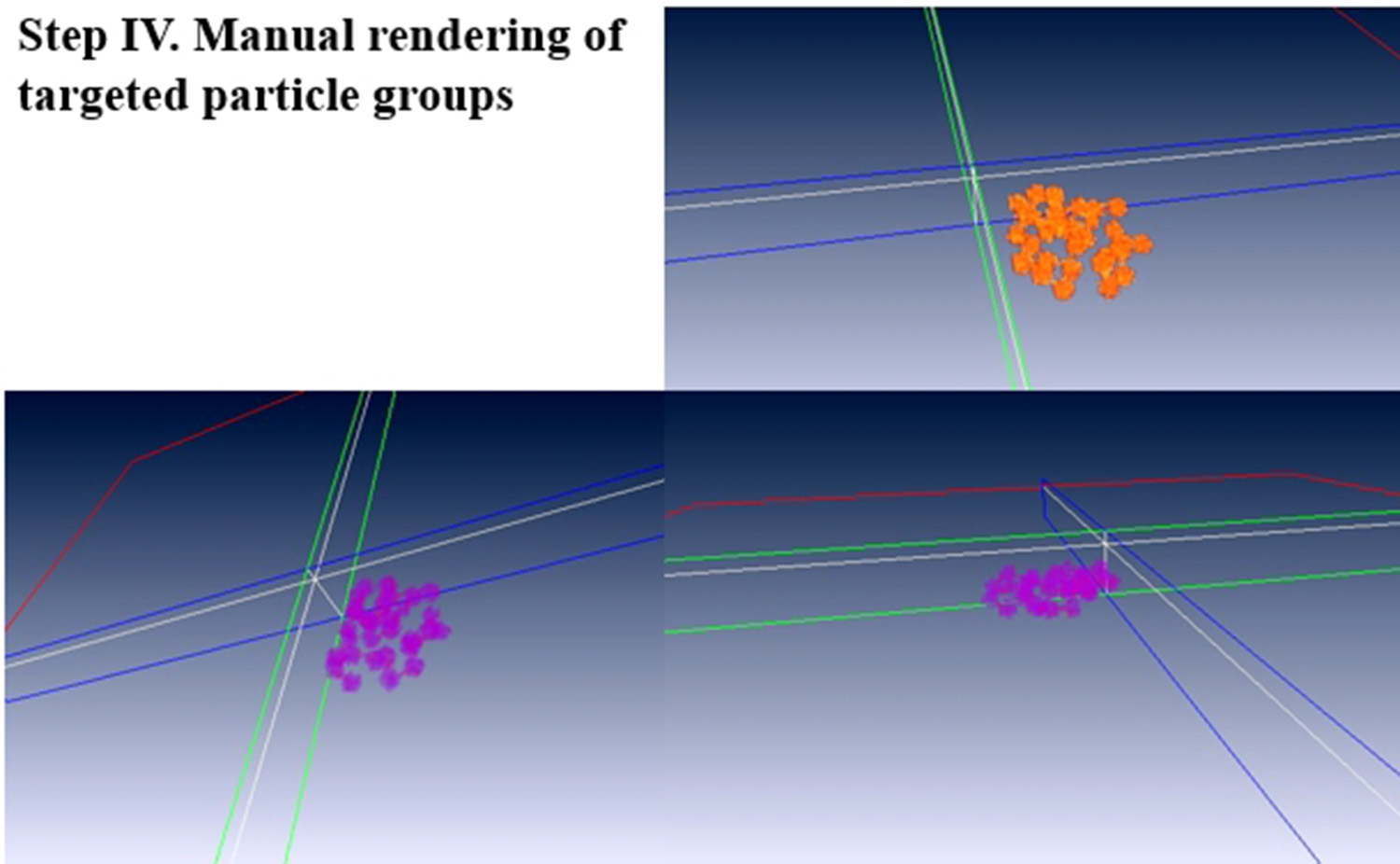

Supplement: Supplementary Information [file srep10790-s1.doc]
